# Expansion of *cagA* Copy Number in *Helicobacter pylori* During Co‐Infection in a Mouse Model

**DOI:** 10.1111/hel.70091

**Published:** 2025-12-29

**Authors:** Kavinda Tissera, Ashansa Ramanayake, Myeong‐A Kim, Eui‐Jeong Noh, Sacheera Angulmaduwa, Dowon Seo, Yong‐Joon Cho, Jeong‐Heon Cha

**Affiliations:** ^1^ Department of Medical Laboratory Science, Faculty of Allied Health Sciences University of Peradeniya Peradeniya Sri Lanka; ^2^ Department of Oral Biology, Oral Science Research Center, Department of Applied Life Science, the Graduate School, BK21 FOUR Project Yonsei University College of Dentistry Seoul Republic of Korea; ^3^ Department of Molecular Bioscience and Multidimensional Genomics Research Center Kangwon National University Chuncheon Republic of Korea

**Keywords:** *cagA* copy number, Co‐infection, *Fusobacterium*, *Helicobacter pylori*, *Salmonella* Typhimurium

## Abstract

**Introduction:**

The virulence factor CagA is critical in mediating inflammation, and *Helicobacter pylori* PMSS1 dynamically modulates *cagA* copy number in response to host immune pressure. Co‐infection with *Salmonella enterica serotype Typhimurium* has been shown to heighten systemic inflammation and reduce *H. pylori* colonization while maintaining type IV secretion system functionality. This suggests that the inflammatory environments induced by co‐infection may influence *H. pylori* virulence mechanisms. This study investigates whether *H. pylori* PMSS1 regulates its *cagA* copy number during systemic co‐infection in a mouse model.

**Methods:**

Across three independent mouse experiments, *H. pylori* isolated from control and co‐infected mice was analyzed for *cagA* gene copy number using quantitative real‐time polymerase chain reaction. Three isolates with the highest *cagA* copy numbers observed to date were further characterized for their virulence phenotypes and subjected to whole‐genome sequencing to identify the mutations associated with co‐infection.

**Results:**

*H. pylori* isolates recovered from the stomachs of co‐infected mice exhibited significantly higher *cagA* copy numbers than those from control mice, with the mean copy number increasing from 2.64 (±1.03) in the control group to 3.22 (±1.63) in the co‐infected group. Three *H. pylori* isolates with high *cagA* copy numbers (6.1, 9.1, and 11.0 copies) were assessed for virulence features. Notably, two of these isolates maintained elevated *cagA* phosphorylation and cell elongation, suggesting their potential relevance as a model for *H. pylori* infection studies in mice. Whole‐genome sequencing further revealed co‐infection‐associated nonsynonymous mutations, notably in the *fur* gene and membrane transport‐related genes such as an MFS transporter and a corrinoid ABC transporter substrate‐binding protein.

**Conclusions:**

These findings suggest that co‐infection with *S.* promotes an increase in the *cagA* copy number, thereby enhancing the virulence potential of *H. pylori* . The study highlights the complex interplay between *H. pylori* and co‐infecting pathogens and their combined impact on *H. pylori* ‐mediated disease, and the utility of high‐*cagA* copy isolates as valuable models for in vivo studies addressing pathogenesis.

**Abbreviations**
*cagA*
: cytotoxin‐associated gene A
*cag*PAI: cytotoxin‐associated gene pathogenicity island
CFU: colony forming units
ELISA: enzyme‐linked immunosorbent assay
FBS: fetal bovine serum

*H. pylori*

:
*Helicobacter pylori*

IL‐8: interleukin 8
PBS: phosphate‐buffered saline
PMSS1: pre‐mouse Sydney strain 1
PVDF: polyvinylidene fluoride
qRT‐PCR: quantitative real‐time polymerase chain reaction
SDS‐PAGE: sodium dodecyl‐sulfate polyacrylamide gel electrophoresis

*S. typhimurium*

:
*Salmonella enterica* serotype typhimurium
T4SS: type IV secretion system

## 1 Introduction

*Helicobacter pylori* infection causes chronic inflammation in the stomach, in some cases leading to gastric pathologies like peptic ulcers or gastric adenocarcinoma, a significant contributor to global cancer mortality [1]. The type IV secretion system (T4SS), encoded by the cytotoxin‐associated gene pathogenicity island (*cag*PAI), plays a pivotal role in disease progression. It translocates various bacterial molecules into host cells, such as the oncogenic protein cytotoxin‐associated gene A (CagA) [2, 3], DNA [4], peptidoglycan [5], and ADP‐heptose [6, 7, 8], which activate inflammatory pathways, leading to chemokines production and sustained chronic inflammation [9, 10].

CagA is one of the major virulence factors driving gastric inflammation and oncogenesis. While many *H. pylori* strains exhibit limited long‐term colonization in murine models, PMSS1 is a well‐characterized clinical isolate that has been extensively used in murine colonization, demonstrating robust in vivo persistence while retaining a fully functional cag pathogenicity island [11]. Recently, we reported for the first time that PMSS1 harbors multiple tandem copies of *cagA* arranged as direct repeats that can dynamically expand or contract even during in vitro passage [12]. In our 2022 study, we extended this finding to in vivo models, where we demonstrated that host immune pressure can drive further dynamic alterations in *cagA* copy number during murine infection, likely to optimize the survival and pathogenicity of the bacterium [13]. In the experiment, PMSS1 recovered from *Rag1* ^*−/−*^ mice, lacking functional T or B cells, retained more *cagA* copies than those from wild‐type mice. Conversely, PMSS1 recovered from *Il10* ^*−/−*^ mice, which display intense inflammation, had fewer *cagA* copies compared to those recovered from wild‐type mice, suggesting that the multi‐*cagA* genotype acts as an immune‐sensitive regulator of *H. pylori* virulence. Thus, PMSS1 offers a unique and tractable system to investigate host‐mediated selection on genomic plasticity.

The idea that one infection can influence the outcome of another through the modulation of the body's innate immune response has been well documented [14, 15, 16, 17]. To further investigate this hypothesis, Skoog et al. [18] developed a co‐infection model to create an environment with an elevated systemic inflammatory response [18]. In the model, mice infected with both *H. pylori* and *Salmonella enterica* serotype Typhimurium ( *S. typhimurium* ) showed heightened inflammation, increased IFN‐γ levels, and reduced *H. pylori* colonization. Despite the reduced bacterial load, *H. pylori* isolates recovered from co‐infected mice retained more effective T4SS functionality than those from mice infected with *H. pylori* alone. Interestingly, co‐infection with *S. typhimurium* influenced T4SS activity, which is crucial for translocating CagA into host cells and mediating CagA‐induced pathogenesis.

Building on these findings, which established a link between co‐infection‐driven inflammation and the regulation of T4SS activity, we aimed to investigate whether *H. pylori* modulates its *cagA* copy number in response to co‐infection. Given that the multi‐*cagA* genotype acts as an immune‐sensitive regulator of *H. pylori* virulence, we sought to understand how *H. pylori* modulates its *cagA* copy number to manage the heightened inflammatory response induced by co‐infection. To address this, we assessed the *cagA* copy numbers of 107 *H. pylori* isolates recovered from control mice and 112 isolates recovered from co‐infected mice. Our results showed that concurrent systemic infection led to an increase in *cagA* copy number, which may enhance *H. pylori* virulence.

## 2 Materials and Methods

### 2.1 Bacterial Strains and Culture Conditions

*H. pylori* PMSS1 was inoculated into C57BL/6J WT mice as described in Skoog et al. [18]. One re‐isolate (HS3‐5.2) from the control group and three re‐isolates (HS1‐9.1, HS1‐10.2, and HS1‐11.6) from the co‐infected group were selected for characterization in AGS cell infection assays. In addition to these strains, PMSS1 wild type, PMSS1 SF‐1 (an isogenic mutant with a single *cagA* copy), PMSS1 ∆*cagA* isogenic mutant and PMSS1 ∆*cagL* isogenic mutant [12] were used for the in vitro AGS cell infection. *H. pylori* culture was maintained as described.

### 2.2 Animals and Experimental Challenge

All animal experiments were conducted at the University of California, Davis, in accordance with NIH guidelines, the Animal Welfare Act and U.S. federal law as described in Skoog et al. [18]. C57BL/6J WT mice were infected with *H. pylori* strain PMSS1 [18]. Briefly, 8‐ to 9‐week‐old female C57BL/6J WT mice were challenged with 1 × 10^9^ colony‐forming units (CFU) of *H. pylori* PMSS1 via oral gavage. For co‐infection experiments, mice received intravenous (iv) injection of 5 × 10^5^ CFU of *S. typhimurium* into the lateral tail vein 1 week after the *H. pylori* infection. At the end of the infection, mice were euthanized, and half of the glandular stomach was homogenized and then plated in serial dilutions onto brucella agar supplemented with 5% heat‐inactivated newborn calf serum and antibiotics. Chromosomal DNA was isolated from single colonies isolated from mice, and the copy number of the *cagA* gene was quantified using quantitative real‐time PCR. Altogether, 219 PMSS1 isolates recovered from 40 mice were examined for *cagA* copy number. Three individual mouse experiments are designated as HS1, HS2, and HS3.

### 2.3 Quantitative Real‐Time Polymerase Chain Reaction (qRT‐PCR)

Real‐time PCR was performed as described in Jang et al. [12]. Briefly, a 145‐bp fragment of *cagA* and a 142‐bp fragment of *ureA* were amplified using specific primers [12]. The relative *cagA* copy numbers were calculated using the 2^−ΔΔCT^ method [19, 20]. *H. pylori* strain PMSS1 SF‐1 [12] possessing single copy of the *cagA* gene was used as a calibrator and *ureA* was used as a reference gene. qRT‐PCR was conducted with SYBR Premix Ex Taq (Tli RNaseH Plus; Takara Bio) on a StepOnePlus Real‐Time PCR system (Life technologies) following the manufacturer's instructions. Data processing was done by StepOneTM software version 2.3 (Life Technologies). For both primer combinations, each sample was assayed in three replicates. Results obtained from different qRT‐PCR runs were calibrated as described by [21]. Mean *cagA* copy numbers were presented with standard deviation in parentheses. A detailed PCR scheme of methodology is provided in the Supporting Information.

### 2.4 Cell Elongation Assay

Cell elongation assay was performed as described in Jang et al. [12]. In brief, AGS cells were seeded in 12‐well culture plates at a density of 2 × 10^5^ cells per well and incubated at 37°C for 22 h. Cells were then starved for 2 h in plain DMEM before infection at a multiplicity of infection (MOI) of 100. At 8 h post‐infection, cells were fixed, and images were taken at 200× magnification using an Olympus CKX41 microscope with a DP20 camera. For each well, 100 randomly selected cells were analyzed. Cell elongation was quantified by calculating the ratio of the longest protrusion length (measured from its tip to the furthest edge of the nucleus) to the nucleus's perpendicular diameter. Measurements were made using ImageJ v1.47 (National Institutes of Health, Bethesda, MD), and average ratios were statistically analyzed. Data for each *H. pylori* strain are presented as box plots, generated using the BoxPlotR tool (http://boxplot.tyerslab.com).

### 2.5 IL‐8 Induction Assay

AGS Cell infection was performed as described in the cell elongation assay. At 8 h post‐infection, cell culture supernatant was collected to measure IL‐8 levels using an enzyme‐linked immunosorbent assay (ELISA). The IL‐8 levels were quantified using a human IL‐8 ELISA Max Deluxe kit (BioLegend, San Diego, CA) according to the manufacturer's instructions. Absorbance was measured using an Epoch microplate reader (BioTek, Winooski, VT). Data are expressed as mean ± SD from three independent replicates.

### 2.6 Immunoblot Assay

To determine the total CagA expression and tyrosine phosphorylation of each *H. pylori* strain, AGS cells seeded onto a 6‐well plate were infected for 8 h. After infection, cells were washed twice with PBS and lysed with 100 μL of cell lysis buffer supplemented with a protease inhibitor cocktail. Total protein concentrations were measured using the Pierce bicinchoninic acid (BCA) protein assay reagent (Thermo Fisher Scientific, Waltham, MA). Twenty micrograms of each sample were separated by 10% SDS‐PAGE and transferred to a 0.45 μm PVDF membrane.

For protein detection, membranes were first incubated with the following primary antibodies: rabbit polyclonal anti‐CagA (b‐300), rabbit polyclonal anti‐UreA (b‐234), rabbit polyclonal anti‐GAPDH, and mouse monoclonal anti‐phosphotyrosine (pY99) for phosphorylated CagA. Membranes were then incubated with HRP‐conjugated secondary antibodies: goat anti‐rabbit and goat anti‐mouse. All primary antibodies were prepared at a 1:10,000 dilution in 3% BSA dissolved in Tris‐buffered saline with 0.1% Tween 20 (TBST). Meanwhile, HRP‐conjugated secondary antibodies were diluted 1: 10,000 in 3% skim milk prepared in TBST. The membranes were developed using WesternBright ECL‐HRP substrate (Advansta, Menlo Park, CA, USA) and visualized on X‐ray film (Agfa, Mortsel, Belgium).

### 2.7 Whole Genome Sequencing, Assembly, and Annotation of Representative Isolates

Using the i‐genomic BYF DNA Extraction Mini Kit (iNTRON Biotechnology), genomic DNA was isolated from representative strains. Following the manufacturer's instructions, library preparation was subsequently carried out with the NEBNext Ultra II FS DNA Library Prep Kit (New England Biolabs). The Illumina NovaSeq X Plus platform was utilized by Genewiz (China) to perform 150 bp paired‐end sequencing. The whole genome sequences are deposited in NCBI GenBank under BioProject accession numbers SRX29923699‐SRX29923702. Alignment of sequencing reads to the PMSS1 reference genome (RefSeq accession: NZ_CP018823.1) was performed using BWA‐MEM v0.7.17. The variants were sorted and indexed using SAMtools v.1.15.1. Variant calling was conducted with FreeBayes (v1.3.10), and high‐confidence SNPs and indels were retained. The resulting variants were then functionally annotated using SnpEff (version 5.2f).

### 2.8 Statistical Analysis

Statistical analyses were conducted using IBM SPSS Statistics version 23 (IBM, Armonk, NY). Differences in *cagA* copy numbers between the control and co‐infected groups were assessed using an independent *t*‐test. Cell elongation and IL‐8 induction data were analyzed using one‐way ANOVA, followed by Tukey's post hoc test for multiple comparisons. *p*‐values were presented along with *F*‐values and degrees of freedom. A *p*‐value < 0.05 was considered statistically significant.

## 3 Results

### 3.1 Enhanced *cagA* Copy Number in *H. pylori* within a Co‐Infected Mouse Model

**FIGURE 1:**
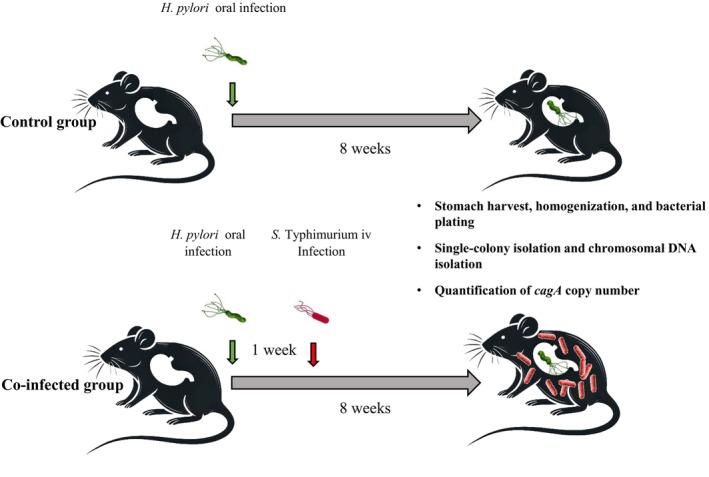
Schematic representation of the mouse infection model. Both control and co‐infected mice were challenged with 1 × 10^9^ CFU of *H. pylori* PMSS1 via oral gavage. One week post‐infection, mice in the co‐infected group were intravenously infected with 5 × 10^5^ CFU of *S. typhimurium* . After 8 weeks of *H. pylori* infection, single‐colony isolates recovered from each mouse were analyzed for *cagA* copy number using qRT‐PCR.

To investigate whether *H. pylori* PMSS1 modulates *cagA* copy number in response to co‐infection with *S. typhimurium* , we examined 219 *H. pylori* isolates from 40 mice across three independent experiments (HS1, HS2, and HS3) conducted at the University of California, Davis. Each experiment included a control group, infected with *H. pylori* alone, and a co‐infected group, infected with both *H. pylori* and *S. typhimurium* (Figure 1). All mice were initially challenged via oral gavage with 1 × 10^9^ CFU of *H. pylori* PMSS1. In the co‐infected group, mice received an intravenous injection of 5 × 10^5^ CFU of *S. typhimurium* 1 week following the *H. pylori* challenge. After 8 weeks of *H. pylori* infection, stomachs were harvested, homogenized, and plated to recover *H. pylori* isolates, which were then analyzed for *cagA* copy number using qRT‐PCR.

In total, we analyzed 219 re‐isolates: 107 from the control group and 112 from the co‐infected group. The co‐infected group consistently exhibited higher *cagA* copy numbers than the control group across all three independent experiments. The mean *cagA* copy number (±SD) across all experiments was 2.64 (±1.03) in control mice, whereas the co‐infected group exhibited a higher mean of 3.22 (±1.63), showing a statistically significant increase (*p* < 0.01). These results strongly suggest that co‐infection with *S. typhimurium* actively promotes the amplification of *cagA* copy numbers in *H. pylori* , potentially contributing to an enhanced virulence phenotype.

### 3.2 HS1 Experiment Shows the Most Pronounced *cagA* Copy Number Increase

The HS1 experiment included 76 *H. pylori* re‐isolates from six control mice (*N* = 35) and nine co‐infected mice (*N* = 41) (Figure S1A). The mean *cagA* copy number in the control group was 2.76 (±1.32), whereas the co‐infected group exhibited a strikingly higher mean of 4.12 (±2.0), marking a statistically significant difference (*p* < 0.01). Additionally, the median *cagA* copy number in the co‐infected group was 3.8, compared to 2.1 in the control group (Figure S1B), further confirming the strong upward shift in *cagA* copy number upon co‐infection. Notably, with the exception of mouse No. 12, most co‐infected mice exhibited higher *cagA* copy numbers than the majority of control mice (Figure S1A), reinforcing the significant *cagA* amplification observed in this experiment.

### 3.3 HS2 and HS3 Experiments Show a Consistent Upward Trend in *cagA* Copy Number

The HS2 experiment included 47 isolates from four control mice (*N* = 23) and five co‐infected mice (*N* = 24) (Figure S2A). While the mean *cagA* copy number increased slightly from 2.52 (±0.82) in the control group to 2.67 (±0.96) in the co‐infected group, the median value was noticeably higher in the co‐infected group (3.3 vs. 2.7 in the control group), suggesting a potential trend toward *cagA* amplification (Figure S2B).

The HS3 experiment involved 48 *H. pylori* isolates from eight control mice and eight co‐infected mice, each contributing six isolates (Figure S3A). The mean *cagA* copy number increased slightly from 2.61 (±0.88) in the control group to 2.70 (±1.08) in the co‐infected group, but no statistically significant difference was observed. However, the median value was again higher in the co‐infected group (3.1 vs. 2.6 in the control group), indicating a possible trend toward increased *cagA* copy numbers (Figure S3B). The variability among individual mice likely contributed to the lack of statistical significance in these two experiments.

### 3.4 Virulence Characteristics of Representative *H. pylori* Re‐Isolates with High *cagA* Copy Number

Several isolates from the co‐infected group exhibited high *cagA* copy numbers that had not been previously documented. We aimed to identify *H. pylori* isolates with elevated *cagA* copy numbers that retained T4SS functionality, as demonstrated by sustained IL‐8 induction and CagA translocation abilities. Identifying these isolates is crucial for advancing research on *H. pylori* infection dynamics and its pathogenic mechanisms.

**FIGURE 2:**
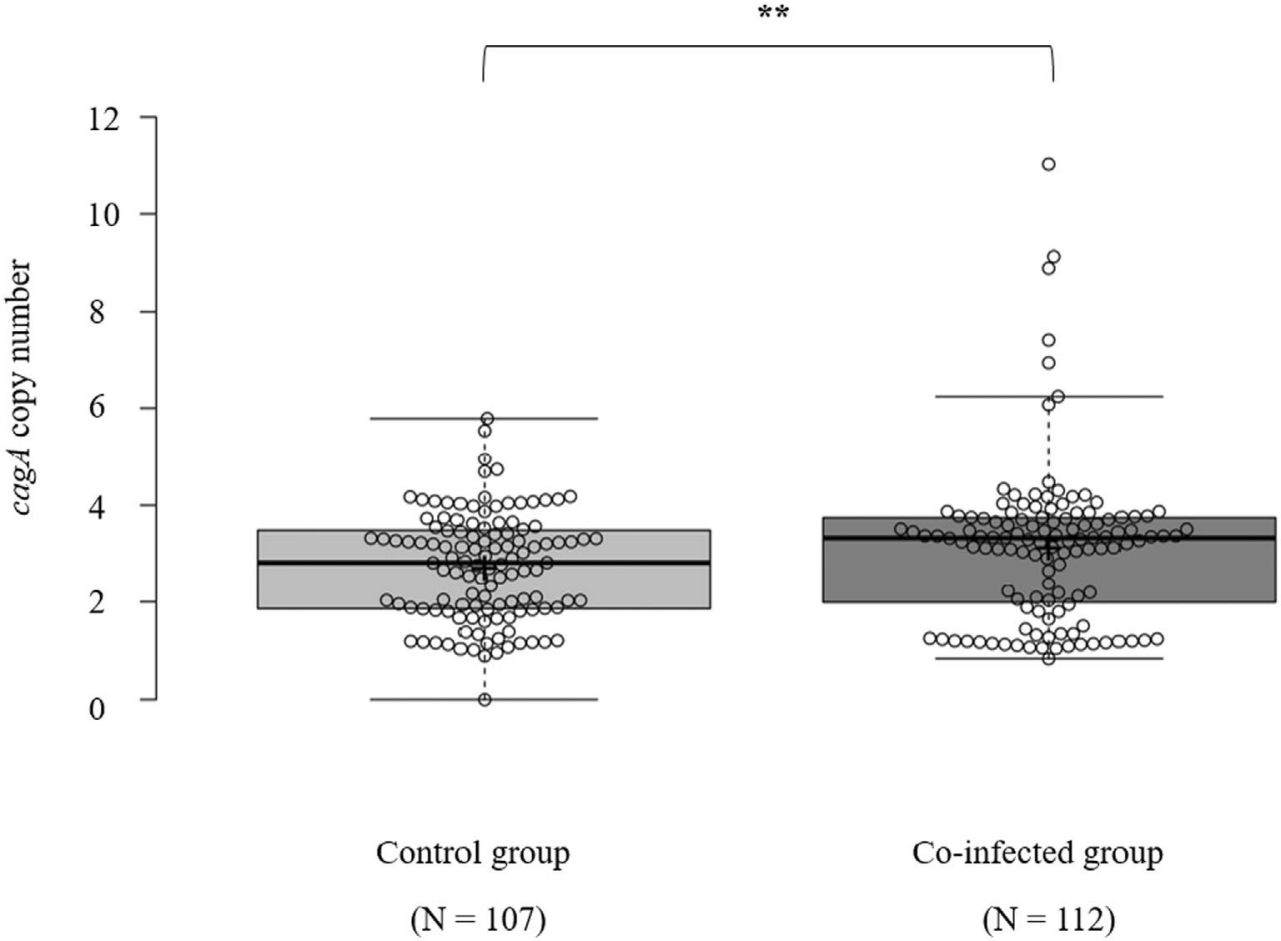
Co‐infection with *S. typhimurium* increases *H. pylori cagA* copies. Box plots depict *cagA* copy numbers from 107 re‐isolates in the control group and 112 re‐isolates in the co‐infected group. Each data point represents a single re‐isolate from respective mice. “**” indicates a significant difference at *p* < 0.01 compared with the control group. The “+” sign denotes the mean *cagA* copy number. The bold central line shows the median, the box limits represent the 25th and 75th percentiles, the whiskers extend to 1.5 times the interquartile range, and outliers appear as separate data points.

We selected three notable isolates carrying the highest copy numbers and functional T4SS from co‐infected mice: HS1‐9.1, HS1‐10.2, and HS1‐11.6, carrying 6.1, 9.1, and 11.0 *cagA* copies, respectively. As a control, we included HS3‐5.2, an isolate from the control group with 3.7 *cagA* copies (Figure 2). Additional controls consisted of PMSS1 wild‐type, PMSS1 SF‐1 (an isogenic mutant with a single *cagA* copy), a *∆cagA* isogenic mutant, and a *∆cagL* isogenic mutant.

### 3.5 IL‐8 Induction

**FIGURE 3:**
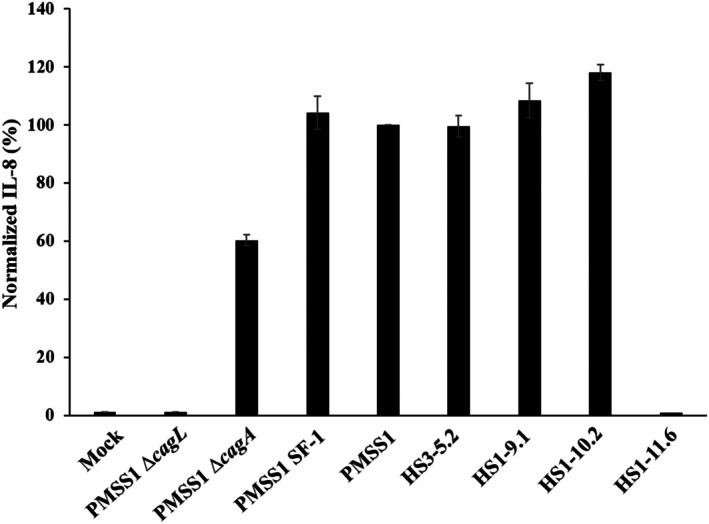
IL‐8 induced by PMSS1 re‐isolates. AGS cells were infected with *H. pylori* strain PMSS1, PMSS1 *∆cagL*, PMSS1 ∆*cagA*, PMSS1 SF‐1, HS3‐5.2, HS1‐9.1, HS1‐10.2 or HS1‐11.6 at a MOI of 100 for 8 h and IL‐8 secretion was determined. Bar graphs illustrate the normalized percentage of IL‐8 secretion by AGS cells for each strain, with error bars representing standard deviation across three independent experiments.

Since T4SS functionality is primarily assessed by IL‐8 induction, we performed an IL‐8 assay to determine if the isolates HS1‐9.1, HS1‐10.2, and HS1‐11.6 had intact T4SS function. AGS cells were infected with these isolates alongside control strains, and IL‐8 levels were measured at 8 h post‐infection (Figure 3). The IL‐8 levels were normalized to those induced by the PMSS1 wild‐type strain.

The isolate HS1‐11.6 exhibited IL‐8 levels comparable to the negative control strain PMSS1 *∆cagL*, indicating a lack of functional T4SS. In contrast, IL‐8 levels induced by HS1‐9.1 and HS1‐10.2 were similar to those of PMSS1, confirming intact T4SS function. Therefore, only HS1‐9.1 and HS1‐10.2 were analyzed further, while HS1‐11.6 was excluded from subsequent studies.

### 3.6 Cell Elongation

**FIGURE 4:**
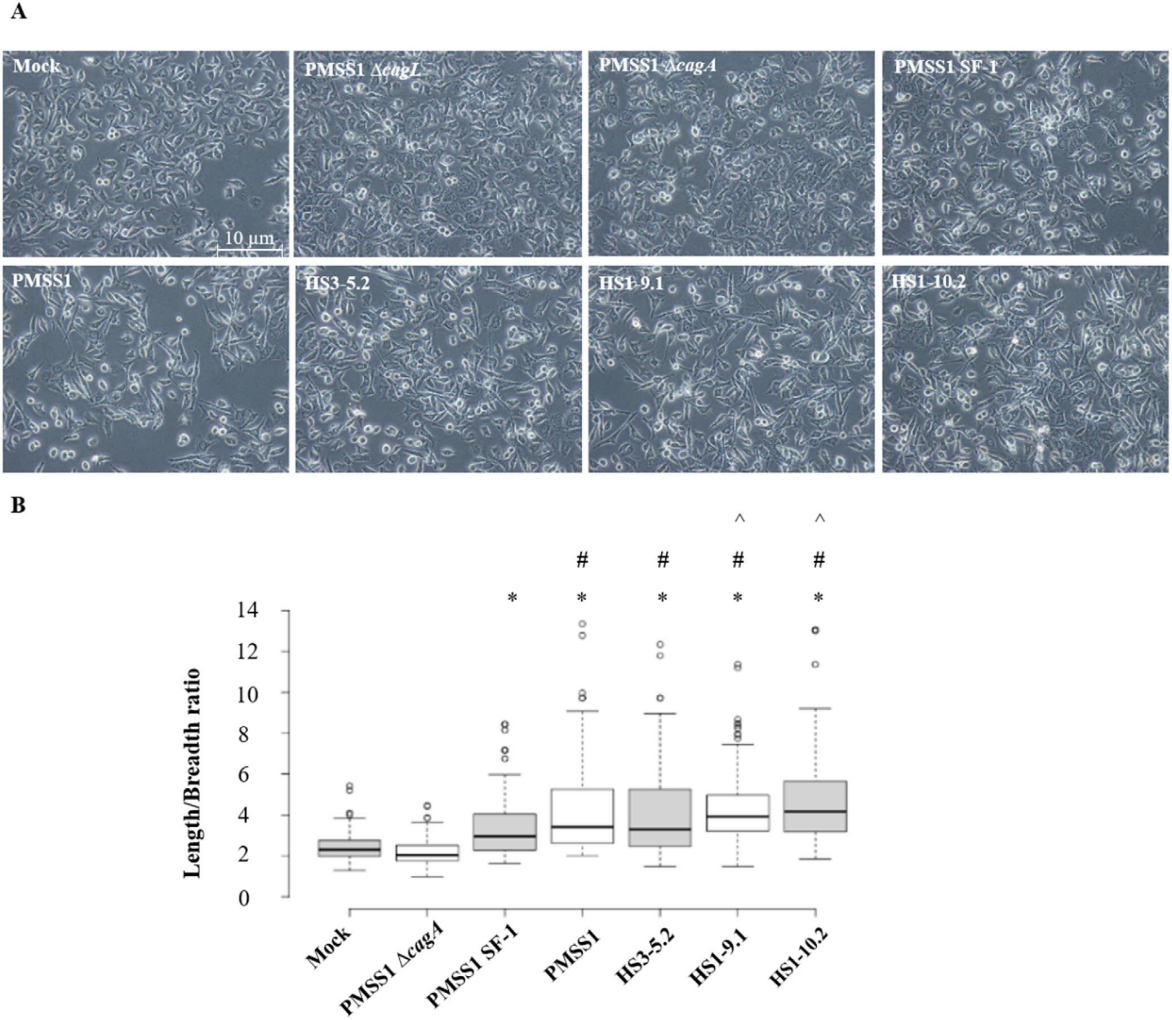
Cell elongation induced by PMSS1 re‐isolates. (A) Representative micrographs of AGS cells infected with different *H. pylori* strains (×200 magnification). (B) Quantification of cell elongation, represented as the length‐to‐breadth ratio. Box plots show bold central line representing medians, the box limits represent the 25th and 75th percentiles, the whiskers extend to 1.5 times the interquartile range, and outliers appear as separate data points. *N* = 100 per group. *, *p* < 0.05 (compared to both the mock‐infected and PMSS1 ∆*cagA* groups); #, *p* < 0.05 (compared to PMSS1 SF‐1, an isogenic mutant carrying single *cagA* gene). ^, *p* < 0.05 (compared to PMSS1 wild type and HS3‐5.2).

Microscopic analysis (Figure 4A) of AGS cell elongation revealed that the PMSS1 SF‐1 strain, containing a single *cagA* gene, induced significant cell elongation compared to the mock control and PMSS1 ∆*cagA* strain. Isolates with multiple *cagA* copies induced greater cell elongation than PMSS1 SF‐1. Specifically, PMSS1 wild‐type and HS3‐5.2, both with 3.7 *cagA* copies, induced more elongation compared to PMSS1 SF‐1. Notably, HS1‐9.1 and HS1‐10.2, possessing 6.1 and 9.1 *cagA* copies, respectively, induced significantly more cell elongation than PMSS1 wild‐type and HS3‐5.2.

Thus, HS1‐9.1 and HS1‐10.2 isolates from the co‐infected group demonstrated more pronounced cell elongation compared to the control group isolate HS3‐5.2 and PMSS1 wild‐type (Figure 4B).

### 3.7 CagA Expression and Translocation

**FIGURE 5:**
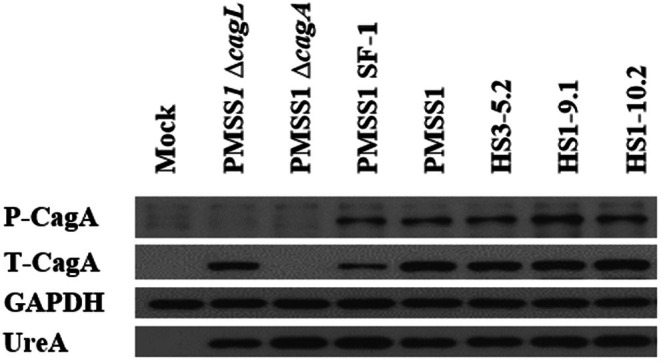
CagA translocation and phosphorylation by PMSS1 re‐isolates. Cell lysates were immunoblotted for phosphorylated CagA (P‐CagA), total CagA (T‐CagA), UreA, and GAPDH. UreA was used as an infection control, while GAPDH was served as a loading control.

To assess CagA expression and CagA translocation, we measured total and phosphorylated CagA levels in isolates HS1‐9.1 and HS1‐10.2 using Western blot analysis. Total CagA expression levels of HS1‐9.1 and HS1‐10.2 were substantially higher than in PMSS1 SF‐1 and comparable to PMSS1 wild‐type (Figure 5). Regarding CagA phosphorylation, HS1‐9.1 exhibited the highest phosphorylation level among all tested strains, indicating enhanced CagA activity in this co‐infected group isolate (Figure 5).

### 3.8 Whole‐Genome Sequencing Reveals Co‐Infection‐Associated Mutations in *H. pylori*

**FIGURE 6:**
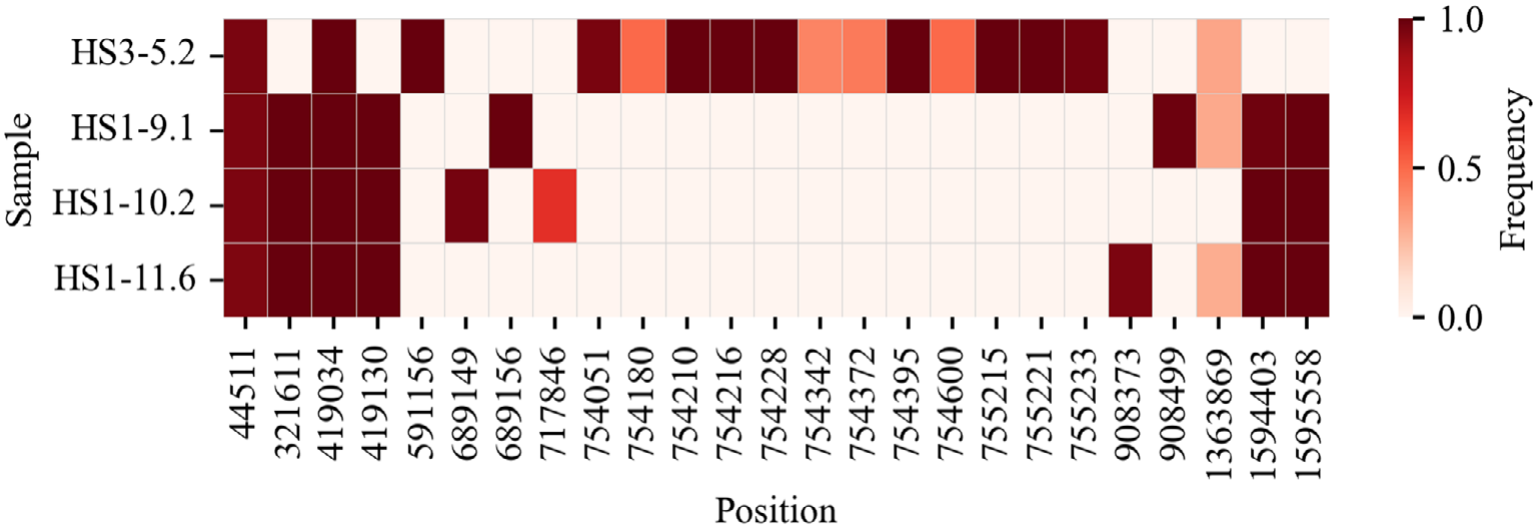
Heatmap of co‐infection‐associated nonsynonymous mutations. The heatmap displays the frequencies of nonsynonymous mutations at genomic positions across four *H. pylori* isolates. Each row represents a distinct isolate, and each column corresponds to a specific genomic position. Color intensity reflects the mutation frequency at each position, ranging from 0 (light color) to 1 (dark color), as indicated by the color scale.

To explore genomic differences associated with co‐infection, we performed whole‐genome sequencing on bacterial strains isolated from co‐infection and control strains. A comparative analysis revealed distinct genetic variations between these groups (Figure 6).

The co‐infection isolates consistently harbored nonsynonymous mutations in the *fur*, an MFS transporter, and a corrinoid ABC transporter substrate‐binding protein genes across all three strains, whereas these mutations were absent in the control isolate (Table S1). This examination suggested that *H. pylori* adopts potential adaptive strategies during in vivo co‐infection, emphasizing the need for future studies to unravel this enigma. Notably, structural rearrangement of the *cagY* gene was detected only in the control isolate, but not in any of the co‐infection isolates, suggesting mechanisms depending on the host inflammatory context.

## 4 Discussion

The interplay between *H. pylori* and host immune responses is complex, particularly in different immune pressures and co‐infections. Our previous in vitro studies have highlighted how *H. pylori* dynamically adjusts its virulence through changes in *cagA* copy number, potentially impacting disease outcomes [12]. In subsequent in vivo studies, we showed that isolates from *Rag1^−/−^* mice, which lack functional T and B cells, retained higher *cagA* copy numbers than those from wild‐type mice, whereas isolates from *Il10^−/−^* mice, which exhibit hyperinflammation, had reduced *cagA* copies [13]. These results suggested that *H. pylori* dynamically adjusts *cagA* copy number in response to immune pressure, likely to optimize fitness and pathogenesis.

Building on this framework, the current study demonstrates that co‐infection with *S. typhimurium* leads to an increase in *cagA* copy number in *H. pylori* PMSS1, which correlates with enhanced virulence, as evidenced by increased CagA phosphorylation and cell elongation. Interestingly, CagA protein levels did not increase proportionally with gene copy number. This observation aligns with our previous report (Jang et al. [12]), where isogenic PMSS1 strains carrying one or four copies of *cagA* exhibited only a twofold increase in CagA protein expression. These findings indicate that gene amplification does not necessarily translate into a linear increase in protein output, due to regulatory layers such as promoter activity, transcriptional repression, mRNA stability, and protein turnover [22]. Moreover, since our assays were performed under in vitro conditions, they may not fully capture the in vivo regulatory environment where variables such as immune signals, metabolic stress, and host cell context may further influence CagA expression. These represent inherent limitations of the current study.

While qPCR enabled us to estimate *cagA* copy number with high sensitivity, direct visualization of large genomic rearrangements remained technically challenging due to the repetitive nature of the *cagA* cluster. In our hands, multiple attempts at long‐range PCR consistently failed, yielding multiple bands likely due to template heterogeneity and polymerase slippage, phenomena that are well documented in repetitive DNA contexts [23]. Moreover, the repetitive structure and large size (≥ 21 kb and up to ~50 kb in high‐copy isolates) of the *cagA* tandem array complicate physical mapping strategies such as PFGE or Southern blotting. These methods are further hindered by the formation of secondary structures and incomplete restriction digestion in repeat‐rich regions, which can result in band smearing and ambiguous hybridization signals [24, 25]. Therefore, among the available approaches, qPCR represented the most reliable and experimentally validated method for quantifying *cagA* copy number, particularly given its previously demonstrated concordance with Southern blotting and the inherent challenges posed by the repetitive nature of the *cagA* locus.

Beyond dynamic changes in the *cagA* gene cluster, our comparative genome sequencing also revealed co‐infection‐specific mutations that may reflect adaptive responses by *H. pylori* . Notably, all three co‐infection isolates shared nonsynonymous mutations in several loci, including genes encoding a major facilitator superfamily (MFS) transporter, a corrinoid ABC transporter substrate‐binding protein, and the *fur* gene, which encodes a global iron‐responsive transcriptional regulator. These mutations were absent in the control isolate. Given the central role of *fur* in regulating virulence, stress response, and metal homeostasis in *H. pylori* , its alteration may represent an adaptive response to the inflammatory and metabolic pressures of co‐infection. Similarly, mutations in membrane transport‐related genes such as the MFS and ABC transporters may reflect shifts in nutrient acquisition, metabolite export, or resistance to host‐derived antimicrobial factors under co‐infection conditions. While the functional consequences of these mutations remain to be investigated, their presence exclusively in co‐infection isolates highlights distinct genomic adaptation pathways shaped by host immune context.

Interestingly, only the control isolate exhibited structural rearrangement of *cagY*, whereas all co‐infection isolates retained an intact *cagY* locus, supporting more stable maintenance of T4SS function under co‐infection conditions. This may suggest that systemic inflammation helps maintain T4SS integrity by suppressing large‐scale recombination in key virulence loci. While the functional implications of these mutations remain to be fully elucidated, they suggest possible shifts in nutrient acquisition or stress resistance mechanisms during co‐infection. These findings are particularly intriguing given the inflammatory and metabolic perturbations associated with *S. typhimurium* co‐infection, and they highlight the potential for systemic immune signals to shape the genomic landscape of colonizing *H. pylori* . Further studies are necessary to determine whether these mutations confer selective advantages under inflammatory conditions or represent early steps in niche‐specific adaptation.

The observed increase in *cagA* copy numbers in co‐infected mice raises a key question: Is this increase driven by *H. pylori* 's intrinsic regulatory mechanisms, or does it result from selective pressure by the host? Notably, the maximum *cagA* copy number in control mice was around 5 (range 0–5), while co‐infected mice exhibited a maximum of 11 (range 1–11). These findings indicate that *H. pylori* strains harboring more than five *cagA* copies were actively generated in response to co‐infection, rather than merely being selected from an existing population. This suggests that regulation of *cagA* copy number plays a dominant role in this process, outweighing the selective elimination of strains with lower *cagA* copies. Nonetheless, it is likely that both regulatory and selective mechanisms contribute to the observed *cagA* amplification in co‐infected mice.

Importantly, we isolated novel high‐copy *cagA* strains (e.g., HS1‐9.1, HS1‐10.2) from co‐infected mice, which retained a functional T4SS and maintained long‐term colonization. These isolates offer valuable tools for dissecting the role of *cagA* amplification in host–pathogen interactions and may inform future studies on gastric disease pathogenesis. Compared to traditional mouse‐adapted strains like SS1, which lack a functional T4SS, these isolates more closely resemble clinically relevant strains and better model human infection dynamics.

Finally, accumulating clinical [26] and experimental evidence supports the idea that co‐infection can exacerbate *H. pylori* virulence and gastric immune pathology. Co‐infection with Epstein–Barr Virus upregulated the *H. pylori* gene expression [27], and *Mycobacterium bovis* aggravated the immune response [28]. Similarly, our findings show that co‐infection alters *cagA* copy number and potentially other genomic features, supporting a broader role for polymicrobial interactions in shaping *H. pylori* pathogenesis.

In conclusion, our study provides strong evidence that systemic co‐infection triggers *cagA* amplification in *H. pylori* , enhancing its virulence potential. These findings underscore the importance of host‐pathogen and pathogen‐pathogen interactions in driving bacterial genome plasticity. Further investigation is warranted to elucidate the molecular mechanisms of *cagA* regulation under inflammatory stress and to explore therapeutic strategies targeting co‐infection‐associated gastric disease progression.

## Author Contributions

**Kavinda Tissera:** validation, investigation, writing – original draft, data curation, formal analysis; **Ashansa Ramanayake:** validation, investigation, writing – original draft, data curation, formal analysis; **Myeong‐A Kim:** resources; **Eui‐Jeong Noh:** resources; **Sacheera Angulmaduwa:** investigation; **Dowon Seo:** data curation, formal analysis; **Yong‐Joon Cho:** data curation, formal analysis; **Jeong‐Heon Cha:** conceptualization, validation, writing – review and editing, supervision, project administration, funding acquisition.

## Conflicts of Interest

The authors declare no conflicts of interest.

## Supporting information


**FIGURE S1:** *cagA* copy number variations of *H. pylori* re‐isolates from HS1 co‐infection experiment. (A) *cagA* copy number variation of re‐isolates in individual mice were illustrated using box plots. (B) Overall mean *cagA* copy numbers of *H. pylori* isolates recovered from the control group (*N* = 35) and the co‐infected group (*N* = 41) are shown in a box plot. Each data point represents a single re‐isolate from the respective mice. “**” indicates a significant difference at *p* < 0.01 compared with control group *H. pylori*. The “+” sign in the box plot denotes the mean *cagA* copy number value. The bold central line represents medians, the box limits represent the 25th and 75th percentiles, the whiskers extend to 1.5 times the interquartile range, and outliers appear as separate data points.
**Figure S2:** *cagA* copy number variation of *H. pylori* re‐isolates from HS2 co‐infection experiment.
(A) *cagA* copy number variations in re‐isolates from individual mice were illustrated using box plots. (B) Overall mean *cagA* copy numbers of *H. pylori* isolates recovered from the control group (*N* = 24) and the co‐infected group (*N* = 23) are shown in a box plot. Each data point represents a single re‐isolate from the respective mice. “NS” indicates no significant difference in *cagA* copy number between the control and co‐infected groups. The “+” sign denotes the mean *cagA* copy number. The bold central line represents medians, the box limits represent the 25th and 75th percentiles, and the whiskers extend to 1.5 times the interquartile range.
**Figure S3:** *cagA* copy number variation of *H. pylori* re‐isolates from HS3 co‐infection experiment. (A) *cagA* copy number variation of re‐isolates from individual mice were illustrated using box plots. (B) Overall mean *cagA* copy numbers of *H. pylori* isolates recovered from the control group (*N* = 48) and the co‐infected group (*N* = 48) are shown in a box plot. Each data point represents a single re‐isolate from the respective mice. The “+” sign indicates the mean *cagA* copy number value. The bold central line represents medians, the box limits represent the 25th and 75th percentiles, and the whiskers extend to 1.5 times the interquartile range.

**Table S1:** Summary of co‐infection‐associated nonsynonymous mutations.

**Data S1:** hel70091‐sup‐0003‐Supinfo.docx.

## Data Availability

The data that support the findings of this study are available from the corresponding author, upon reasonable request.
