# Supplementary material for: Expansion of cagA Copy Number in Helicobacter pylori During Co‐Infection in a Mouse Model
Source: Helicobacter. 2025 Dec 29;30(6):e70091. doi: 10.1111/hel.70091 (PMC12748022; doi:10.1111/hel.70091)
Supplement: Supplementary file 1 — FIGURE S1: cagA copy number variations of H. pylori re‐isolates from HS1 co‐infection experiment. (A) cagA copy number variation of re‐isolates in individual mice were illustrated using box plots. (B) Overall mean cagA copy numbers of H. pylori isolates recovered from the control group (N = 35) and the co‐infected group (N = 41) are shown in a box plot. Each data point represents a single re‐isolate from the respective mice. “**” indicates a significant difference at p < 0.01 compared with control group H. pylori. The “+” sign in the box plot denotes the mean cagA copy number value. The bold central line represents medians, the box limits represent the 25th and 75th percentiles, the whiskers extend to 1.5 times the interquartile range, and outliers appear as separate data points. Figure S2: cagA copy number variation of H. pylori re‐isolates from HS2 co‐infection experiment. (A) cagA copy number variations in re‐isolates from individual mice were illustrated using box plots. (B) Overall mean cagA copy numbers of H. pylori isolates recovered from the control group (N = 24) and the co‐infected group (N = 23) are shown in a box plot. Each data point represents a single re‐isolate from the respective mice. “NS” indicates no significant difference in cagA copy number between the control and co‐infected groups. The “+” sign denotes the mean cagA copy number. The bold central line represents medians, the box limits represent the 25th and 75th percentiles, and the whiskers extend to 1.5 times the interquartile range. Figure S3: cagA copy number variation of H. pylori re‐isolates from HS3 co‐infection experiment. (A) cagA copy number variation of re‐isolates from individual mice were illustrated using box plots. (B) Overall mean cagA copy numbers of H. pylori isolates recovered from the control group (N = 48) and the co‐infected group (N = 48) are shown in a box plot. Each data point represents a single re‐isolate from the respective mice. The “+” sign indicates the mean cag [file HEL-30-e70091-s001.docx]

**Supplementary Figures**

**
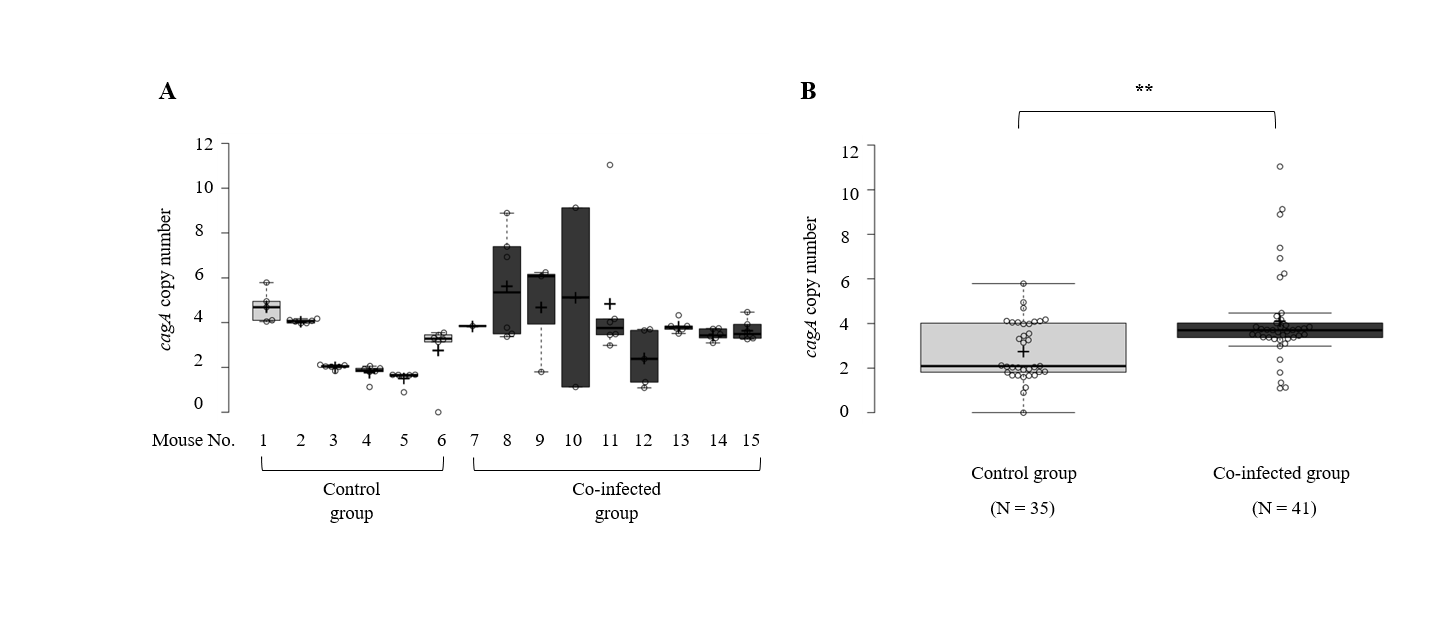
**

**Fig. S1 *cagA* copy number variations of *H. pylori* re-isolates from HS1 co-infection experiment.**

(A) *cagA* copy number variation of re-isolates in individual mice were illustrated using box plots. (B) Overall mean *cagA* copy numbers of *H. pylori* isolates recovered from the control group (N = 35) and the co-infected group (N = 41) are shown in a box plot. Each data point represents a single re-isolate from the respective mice. "**" indicates a significant difference at *p* < 0.01 compared with control group *H. pylori*. The "+" sign in the box plot denotes the mean *cagA* copy number value. The bold central line represents medians, the box limits represent the 25^th^ and 75^th^ percentiles, the whiskers extend to 1.5 times the interquartile range, and outliers appear as separate data points.


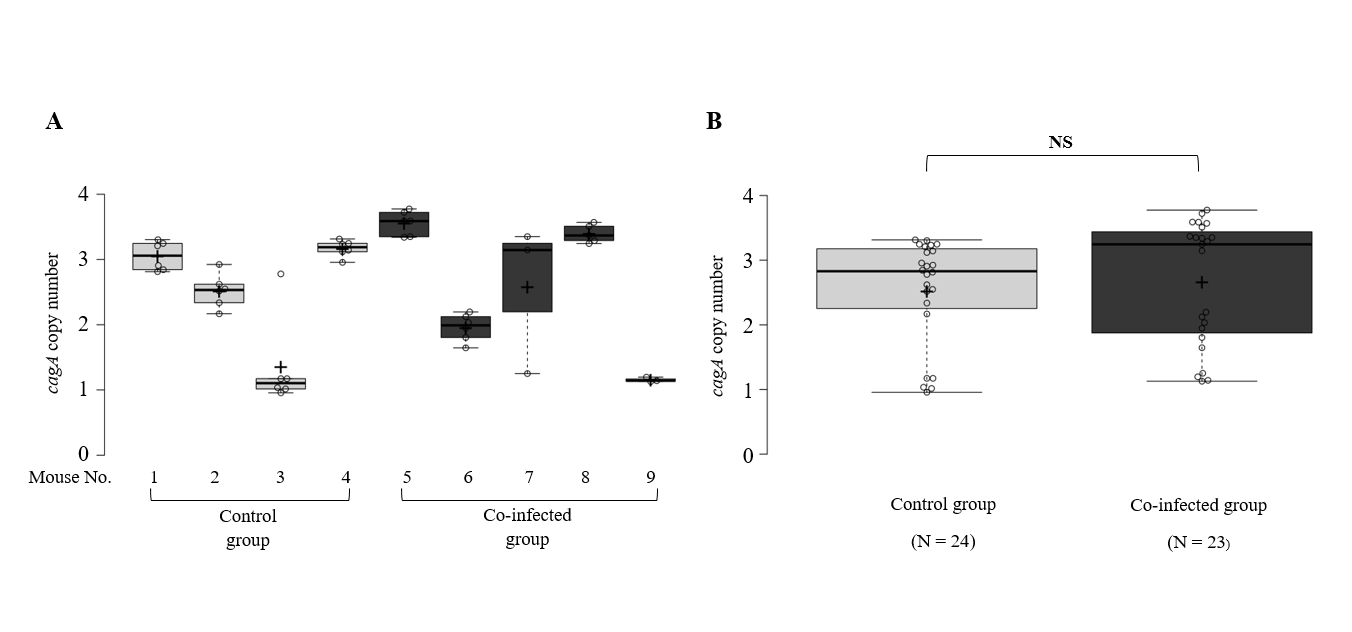


**Fig. S2 *cagA* copy number variation of *H. pylori* re-isolates from HS2 co-infection experiment.**

(A) *cagA* copy number variations in re-isolates from individual mice were illustrated using box plots. (B) Overall mean *cagA* copy numbers of *H. pylori* isolates recovered from the control group (N = 24) and the co-infected group (N = 23) are shown in a box plot. Each data point represents a single re-isolate from the respective mice. “NS” indicates no significant difference in *cagA* copy number between the control and co-infected groups. The “+” sign denotes the mean *cagA* copy number. The bold central line represents medians, the box limits represent the 25^th^ and 75^th^ percentiles, and the whiskers extend to 1.5 times the interquartile range.


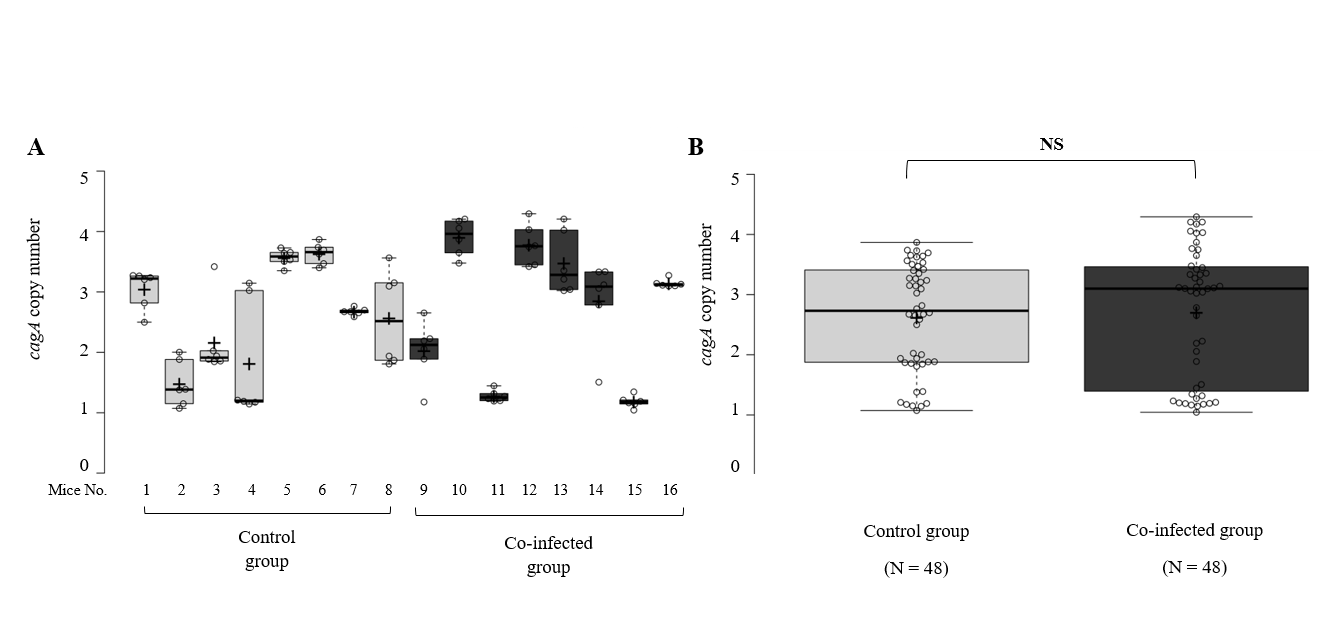


**Fig. S3 *cagA* copy number variation of *H. pylori* re-isolates from HS3 co-infection experiment.** (A) *cagA* copy number variation of re-isolates from individual mice were illustrated using box plots. (B) Overall mean *cagA* copy numbers of *H. pylori* isolates recovered from the control group (N = 48) and the co-infected group (N = 48) are shown in a box plot. Each data point represents a single re-isolate from the respective mice. The “+” sign indicates the mean *cagA* copy number value. The bold central line represents medians, the box limits represent the 25^th^ and 75^th^ percentiles, and the whiskers extend to 1.5 times the interquartile range.
