# Supplementary material for: Expansion of cagA Copy Number in Helicobacter pylori During Co‐Infection in a Mouse Model
Source: Helicobacter. 2025 Dec 29;30(6):e70091. doi: 10.1111/hel.70091 (PMC12748022; doi:10.1111/hel.70091)
Supplement: Supplementary file 3 — Data S1: hel70091‐sup‐0003‐Supinfo.docx. [file HEL-30-e70091-s002.docx]

**Real-Time PCR Scheme for Quantification of *cagA* Copy Number**

**1. Target Region and Primer Design**

A 12,374-bp region of PMSS1 was mapped based on the DNA sequence of the *cagA* genes and their flanking regions. Primer annealing sites used for PCR (filled triangles) are indicated. Three repeated *cagA* homologous areas (CHAs) were designated CHA-ud (red), CHA-u (yellow), and CHA-d (green).

RTcagAR

RTcagAF

RTcagAF

RTcagAR


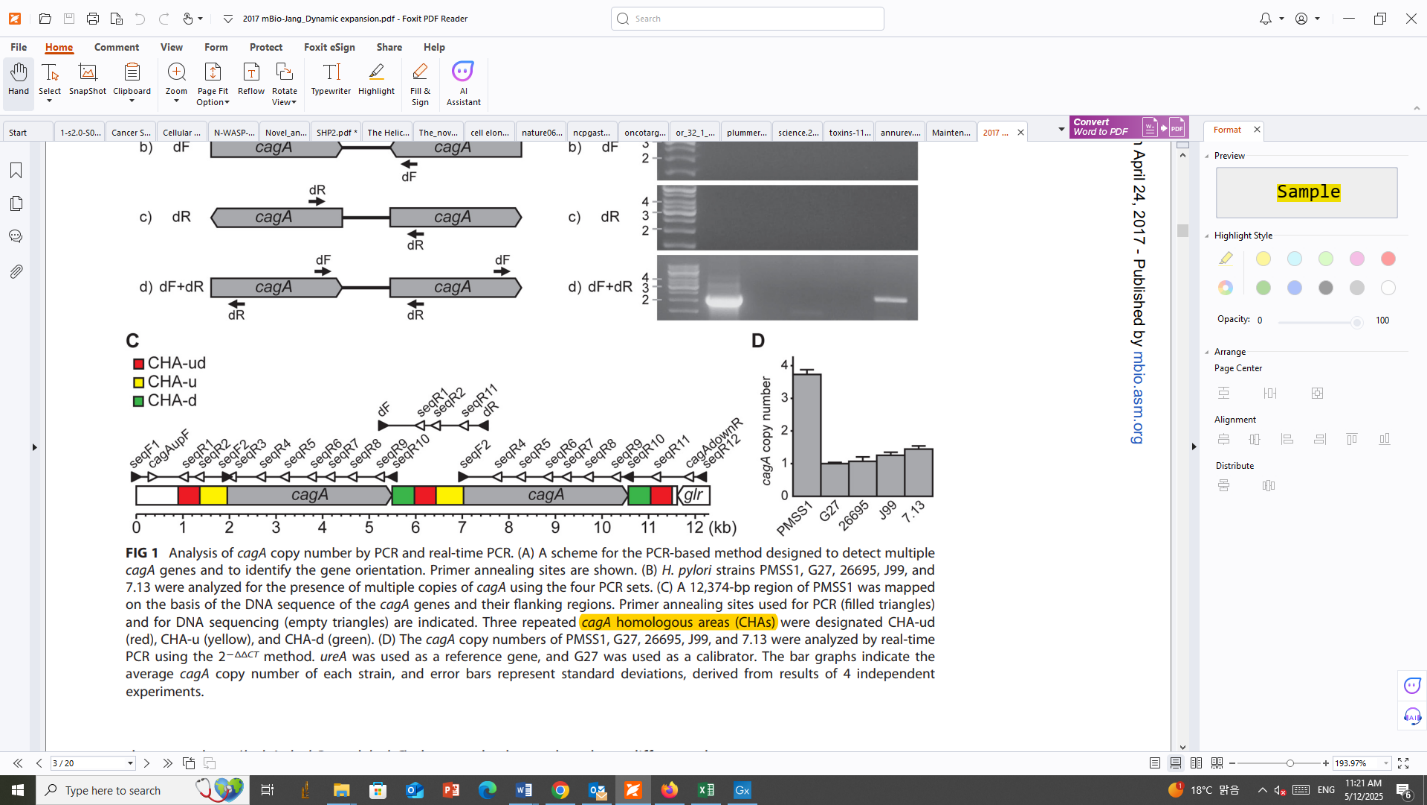


**2. Primer Sequences**

Gene-specific primers were used to amplify a 145-bp fragment of *cagA* and a 142-bp fragment of the housekeeping gene *ureA* (used as an internal reference). Primer sequences and target coordinates (based on the PMSS1 genome) are provided below:

| Primer name | Sequence (5′→3′) | Description |
| --- | --- | --- |
| RTcagAF | CCC TTA AAG GCT CGG TGA A | PMSS1 *cagA* orf 2,162-2,180. |
| RTcagAR | TTT TCA AGG TCG CTT TTT GC | PMSS1 *cagA* orf 2,287-2,306. |
| RTureAF | AAA AGC CGT TAG CGT GAA AGT | PMSS1 *ureA* orf 372-392. |
| RTureAR | CCC GCT CGC AAT GTC TAA G | PMSS1 *ureA* orf 495-513. |

**3. Real-Time PCR Conditions**

Amplification was carried out using the SYBR® Premix Ex Taq™ with ROX reference dye (TaKaRa, Japan) on a StepOnePlus™ Real-Time PCR System (Life Technologies). Each reaction was performed in triplicate. Data processing was performed using StepOneTM software version 2.3 (Life Technologies). Fluorescence was measured at the end of 60°C step in each cycle. Melting curve analysis confirmed specificity of the amplified products.

| PCR Stage | PCR step | PCR parameters | Cycles |
| --- | --- | --- | --- |
| Stage 1 | Initially denaturation | 95°C for 30 s | 1 |
| Stage 2 | PCR for 40 cycles | 95°C for 5s | 40 |
|  |  | 60°C for 30s |  |
| Stage 3 | Dissociation stage | 95°C for 15s | 1 |
|  |  | 60°C for 60s |  |
|  |  | 95°C for 15s |  |

**4. Data Analysis and Normalization**

Amplification efficiencies of *cagA* and *ureA* were calculated using a 10-fold serial dilution of *H. pylori* chromosomal DNA. These efficiencies were incorporated into the inter-plate calibration using the method described by Hellemans et al., 2007.

Relative *cagA* gene copy number was calculated using the 2^-ΔΔCT method with the following parameters:

• ΔCT_sample = CT (*cagA*) − CT (*ureA*)

• ΔCT_calibrator = CT (*cagA*, PMSS1/cagA-SF-1) − CT (*ureA*, PMSS1/cagA-SF-1)

• ΔΔCT = ΔCT_sample − ΔCT_calibrator

The PMSS1/*cagA*-SF-1 strain (harboring a single *cagA* copy) served as the calibrator.

An example for a calculation is provided below.

| **strain** | **CagA Ct** | **Average** | **UreA Ct** | **Average** | **∆CT (CT *cagA* – CT *ureA*) (Normalization)** | **CagA ratio**  **(1/2^∆CT)** | **Relative *cagA* copy number** |
| --- | --- | --- | --- | --- | --- | --- | --- |
| PMSS1 SF-1 | 15.61 | 15.44 | 15.85 | 15.80 | -0.36 | 1.28 | 1 |
|  | 15.29 |  | 15.82 |  |  |  |  |
|  | 15.42 |  | 15.72 |  |  |  |  |
| G27 | 15.79 | 15.71 | 15.88 | 15.85 | -0.14 | 1.10 | 0.9 |
|  | 15.72 |  | 15.82 |  |  |  |  |
|  | 15.63 |  | 15.84 |  |  |  |  |
| PMSS1 | 13.76 | 13.63 | 15.96 | 15.82 | -2.19 | 4.57 | 3.6 |
|  | 13.58 |  | 15.69 |  |  |  |  |
|  | 13.54 |  | 15.80 |  |  |  |  |
